# Enzyme Molecules in Solitary Confinement

**DOI:** 10.3390/molecules190914417

**Published:** 2014-09-12

**Authors:** Raphaela B. Liebherr, Hans H. Gorris

**Affiliations:** Institute of Analytical Chemistry, Chemo- and Biosensors, University of Regensburg, Regensburg 93040, Germany

**Keywords:** enzyme kinetics, single molecule enzymology, fluorescence microscopy, femtoliter array

## Abstract

Large arrays of homogeneous microwells each defining a femtoliter volume are a versatile platform for monitoring the substrate turnover of many individual enzyme molecules in parallel. The high degree of parallelization enables the analysis of a statistically representative enzyme population. Enclosing individual enzyme molecules in microwells does not require any surface immobilization step and enables the kinetic investigation of enzymes free in solution. This review describes various microwell array formats and explores their applications for the detection and investigation of single enzyme molecules. The development of new fabrication techniques and sensitive detection methods drives the field of single molecule enzymology. Here, we introduce recent progress in single enzyme molecule analysis in microwell arrays and discuss the challenges and opportunities.

## 1. Introduction

Enzymes are omnipresent catalysts of biochemical reactions. Detailed studies of enzymes and their catalytic activity have provided us with a global understanding of enzyme structure and functionality. Currently, our knowledge about enzyme kinetics mainly relies on bulk-phase experiments [1,2,3], in which the activity of a large number of molecules—in the range of 10^12^–10^18^ or higher—is measured and the kinetic parameters are averaged over the whole population. Therefore, no information about the contribution of individual enzyme molecules can be gained. The development of single molecule technologies has provided new insights into enzyme catalysis that was previously hidden in bulk reactions. [4,5] Most single molecule experiments are based on fluorescence technologies, which are highly sensitive because–depending on the type of fluorophore–one molecule can emit up to 10^6^ photons before it eventually photobleaches. Fluorescence microscopy has become an essential method for the non-invasive interrogation of biomolecules, invigorated by new methods to increase the optical resolution of microscopy beyond the diffraction limit of light. Today, fluorescence microscopy is applied to study the motion and interaction of individual molecules, molecular cooperativity or protein folding.

Fluorescence microscopy can also visualize the substrate turnover of individual enzyme molecules, unravelling broad activity distributions and dynamic fluctuations within enzyme populations [6]. Different conformational states of individual enzyme molecules entail dynamic fluctuations such as varying substrate turnover rates over time (dynamic heterogeneity) [7,8,9,10] or broad distributions of catalytic rates within an enzyme population (static heterogeneity) [11,12,13]. A microscopic view on enzyme reactions has disclosed differences between individual enzyme molecules in a population and provided us with a deeper understanding of enzyme-substrate interactions as well as enzyme kinetics.

The single molecule approach to enzyme kinetics requires a reformulation of the classical Michaelis-Menten equation [1]. In the simplest case, an enzyme reaction (e.g., a hydrolytic reaction) can be formulated as:
(1)
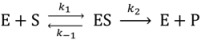


An enzyme (E) binds a substrate (S) to form an enzyme-substrate complex (ES) with a rate constant k_1_. (ES) can either dissociate with the rate constant *k*_−1_ or convert to the free enzyme (E) and product (P) in an irreversible step (*k*_2_). The velocity of an enzyme reaction is proportional to the concentration of the ES complex:
(2)
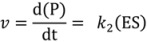


In traditional enzymatic ensemble measurements a constant concentration of (ES) is assumed (steady-state assumption) and the enzymatic velocity is defined as:
(3)
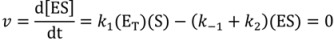


Rearranging and considering (E_T_) = (E) + (ES) yields the Michaelis-Menten equation:
(4)
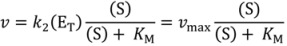


*v*_max_ is the maximum enzymatic turnover rate at substrate saturation and *K*_M_ = (*k*_−1_ + *k*_2_)/*k*_1_ is the substrate concentration where the enzymatic velocity is half of *v*_max_. *K*_M_ and *v*_max_ can be calculated by measuring v as a function of the substrate concentration.

In a single-molecule experiment, however, the conventional steady-state assumption is not valid anymore, as a single enzyme molecule is either bound in the complex (ES) or is free (E). Instead, the concentration (ES) is replaced by the probability of finding the enzyme molecule in its bound state δ(ES) [5,11,14]. δ(ES) is given by a modified Michaelis-Menten equation without the restrictive condition of (S) >> (E):
(5)
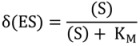


The velocity of a reaction catalyzed by an individual enzyme molecule can thus be defined as:
(6)
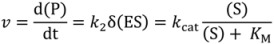


Other expressions of the Michaelis-Menten equation have also been derived to account for the different observables in single enzyme molecule experiments. For example, the group of Xie [10,15] observed the generation of individual product molecules in an enzyme reaction by monitoring the emission of fluorescent bursts originating from each substrate turnover event. The reaction rate of an individual enzyme was evaluated from the inverse of the mean waiting time 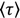 between two successive turnover events. In this case, the Michaelis-Menten equation was reformulated to:
(7)
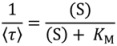


The enzyme reaction, however, is considerably more complex than indicated by Equations (6) and (7): As mentioned above, single molecule experiments have revealed static and dynamic disorder within an enzyme population. Therefore a comprehensive Michaelis-Menten model needs to be extended to include conformational fluctuations and their impact on enzyme kinetics [7,16]. In the fluctuating-enzyme model, it is assumed that enzymes change between different conformational states, each with its own characteristic kinetic parameters. To take this into account, the classical Michaelis-Menten model can be extended by a thermodynamic “dimension”. A detailed derivation of the extended version of the MM-model, considering not only varying enzymatic states but also other factors such as memory effects from substrate imprinting can be found in publications by Min *et al*., and Claessen *et al*. [17,18,19].

Technologies for single molecule enzymology have disclosed fascinating kinetic details. Additionally, they have uncovered enzymatic subpopulations hidden in bulk-phase experiments and enabled the assessment of cooperativity in oligomeric enzymes such as β-galactosidase [20]. Moreover, one can obtain information on the impact of external influences such as temperature [21,22,23,24,25], pH, inhibitors [26,27] or buffer additives [28] on enzymatic reactions. In summary, the development of more sensitive detection methods and new single molecule technologies has greatly extended our understanding of the fundamental biochemical processes of life.

## 2. Techniques for the Analysis of Single Protein Molecules

Biological systems have been studied by using a broad range of single molecule techniques that have emerged over the last twenty years. Generally, two fundamentally different forms of single-molecule experiments can be distinguished. The first type is based on the detection of single fluorophore molecules to monitor single-molecule dynamics [29,30,31], single-molecule trajectories [32] and conformational changes [33,34,35]. A wide range of such single fluorophore detection techniques [36,37,38,39,40] allow for studying structural and behavioral diversities between individual biomolecules. For example, single enzymatic turnover events can be observed by recording bursts of fluorophores released in subsequent catalytic cycles [10]. For detecting single fluorophore molecules, however, it is necessary to minimize the high background fluorescence originating from inelastic (Raman) and elastic (Rayleigh) scattering of surrounding molecules and fluorescent contaminations [41]. The key to background reduction is to keep the excitation or detection volume as small as manageable, for example by using total internal reflection fluorescence (TIRF) microscopy [42,43,44] or confocal microscopy [5,6,7,8,10,16,45,46,47,48]. There are many excellent reviews on single molecule experiments that rely on the detection of single-turnover events [49,50,51,52,53,54,55,56].

This review focuses on the second type of single enzyme molecule experiments that can investigate the catalytic activity of individual enzyme molecules without the need for detecting single fluorophore molecules. In traditional bulk experiments, the activity of enzymes is usually determined from the increase of product concentration over time. This principle can also be applied to single molecule experiments. An individual enzyme molecule is isolated with a fluorogenic substrate in a defined volume and the accumulation of the fluorescent product can be monitored over time. Owing to the small reaction volume (usually between 10 to 1000 fL) a small amount of accumulated fluorescent product is sufficient to exceed the limit of detection.

**Figure 1:**
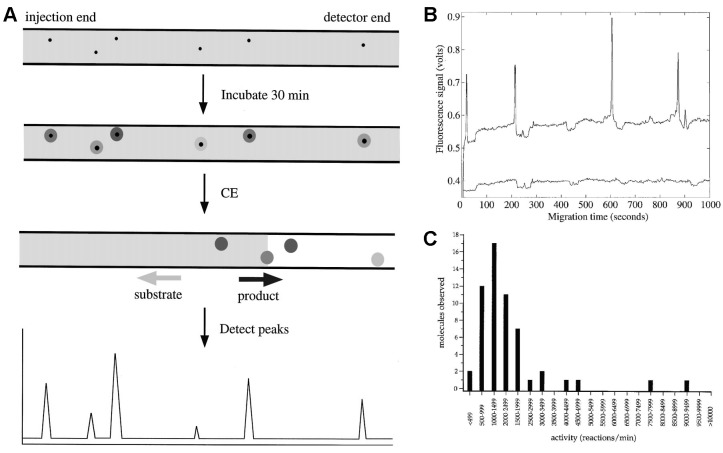
Single molecule enzymology by capillary electrophoresis. (**A**) The capillary is filled with a solution of substrate (grey) and highly diluted enzyme (black dots). After incubation the product accumulates in the vicinity of the enzyme molecules. When an electric field is applied, the substrate migrates towards the injection-end whereas the product migrates towards the detector, generating peaks in the electropherogram; (**B**) electropherogram of the single molecule β-galactosidase assay; (**C**) the substrate turnover distribution of single β-galactosidase molecules indicates a broad conformational heterogeneity within the enzyme population. Modified schematic representation reprinted with permission from Canadian Science Publishing [74], © 1998.

Single enzyme molecules can be isolated and investigated free in solution by using capillary electrophoresis in combination with laser-induced fluorescence (CE-LIF) (Figure 1). CE enables the separation of substrate and product of an enzymatic reaction based on their different electrophoretic mobility. Thus, the formation of a fluorescent product and the loss of the substrate can be monitored in parallel. A very dilute enzyme solution is filled into a narrow capillary together with the fluorogenic substrate. At low enzyme concentrations each molecule is separated by several centimeters such that the diffusion zones of the individual enzyme molecules do not overlap. The local accumulation of fluorescent product can be attributed to a single enzyme molecule. Each product zone migrates to the detector where it is monitored. The peak area is proportional to product formation. CE-LIF for single enzyme molecule analysis was first introduced by Xue and Yeung in 1995 [12] and further developed in the groups of Dovichi and Craig. The technique has been successfully applied for detecting and analyzing individual molecules of β-galactosidase [23,57,58,59,60,61,62,63], alkaline phosphatase [24,64] and lactate dehydrogenase [12]. CE confines a volume in only two directions but is open in the flow direction. Therefore, it is usually not suitable for time-resolved measurements.

**Figure 2:**
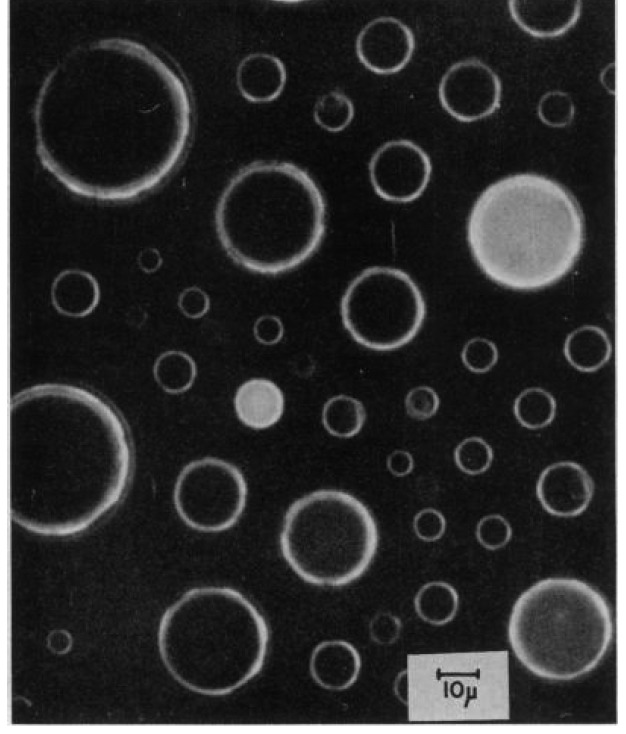
Photograph of the first single enzyme molecule experiment performed by Rotman in water in oil emulsion droplets. Individual molecules of β-galactosidase together with a fluorogenic substrate are enclosed in the water droplets. The enzymatic substrate turnover of single enzyme molecules is monitored by the generation of fluorescent product, which appears white in the image. Image reprinted with permission from [21].

The three-dimensional isolation of individual enzyme molecules in separate compartments allows for performing time-resolved measurements to investigate single enzyme molecule dynamics. The first isolation of individual enzyme molecules and their substrate in a confined volume was performed by Rotman *et al.* [21], who enclosed individual β-galactosidase molecules in droplets of a water-in-oil emulsion (Figure 2). To date, individual biomolecules have been isolated by using various types of emulsion-defined femtoliter droplets [13,65,66,67,68,69,70,71,72]. To avoid the generation of multi-enzyme droplets, the enzyme is highly diluted before the emulsion is generated. As the volume of droplets prepared by standard emulsification techniques is broadly distributed, additional steps have frequently been used to exclude larger droplets from data analysis [73].

**Figure 3:**
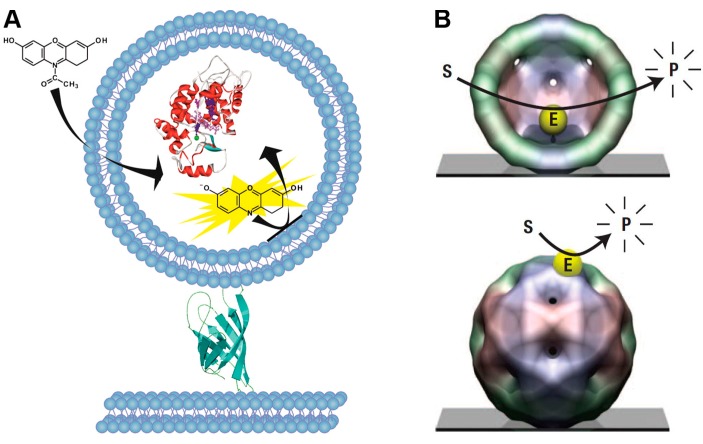
Enclosure of individual enzyme molecules in ultra-small, self-assembled reaction chambers. (**A**) An individual HRP molecule is encapsulated within a large unilamellar vesicle. The externally added substrate Amplex Red diffuses into the vesicle and is oxidized by HRP to the fluorescent product resorufin, which remains trapped in the vesicle interior; (**B**) A single HRP molecule (E) is confined in an icosahedral virus capsid. The fluorogenic substrate (S) penetrates the capsid where it is converted to fluorescent product (P). The accumulated product is monitored by fluorescence microscopy before it finally diffuses out through the capsid pores. Modified schematic representation reprinted with permission from [76] and from Macmillan Publishers [80], © 2010.

Other ultra-small reaction chambers such as lipid vesicles, virus capsids or even living cells have also been successfully applied for the confinement of individual biomolecules. Liposomes or lipid vesicles define a small reaction volume enclosed by lipid membranes, where both bulk phase and vesicle content are aqueous solutions (Figure 3A) [75,76,77]. There are a broad range of methods for preparing liposomes as reviewed by Jesorka and colleagues [78]. Liposomes can confine volumes similar in size as bacterial cells and are thus well suited for the investigation of biological processes [79]. For enclosing single enzyme molecules in virus capsids, usually the cowpea chlorotic mottle virus (CCMV) has been employed that provides a small reaction chamber in form of an icosahedral protein capsid (Figure 3B) [80,81,82]. Virus capsids have an inner diameter of several nm, defining a volume of a few zeptoliter (zL). To overcome the limitations of the ultra-small confinement, a single enzyme molecule is enclosed inside the virus capsid, whereas substrate and product can pass by diffusion due to the size-selective permeability of the protein cage [82]. Similar as liposomes or virus capsids, living cells can confine single biomolecules [83]. Single molecule studies in living cells can provide new insights into fundamental biochemical processes in a physiological environment [84,85,86].

Spatial confinement of enzyme molecules in small reaction containers avoids the need for immobilizing the enzyme on a surface, which can lead to steric hindrance, partial inactivation or disturbance of the original enzyme activity [6]. Self-assembled micro-vessels for single enzyme molecule analysis (bottom-up approach) [87] such as water droplets in oil, lipid vesicles or virus capsids can have variable sizes and need to be surface immobilized for long-time monitoring. These problems of single enzyme molecule analysis can be avoided by the systematic structuring of surfaces (top-down approach) [87] to generate large arrays of thousands of reaction chambers with uniform size and defined position.

## 3. Single Molecule Enzymology in Femtoliter Arrays

Homogeneous arrays of femtoliter-sized reaction vessels can be fabricated in the surface of optical-fiber bundles, glass coverslips, poly(dimethylsiloxane) (PDMS) sheets or thermoplastics by using established microfabrication techniques. The microchambers usually define a volume of several femtoliters (fL) with a diameter between 3 µm and 10 µm and a depth between 200 nm and 5 µm [88,89,90]. Because of the small dimensions of the fl-containers, they can be arranged in very high density arrays [91]. For example, fl-arrays etched into the surface of a glass coverslip have a density of 10,000 mm^−2^ [92]. When these arrays are filled with an enzyme solution, thousands of individual molecules can be observed in parallel, enabling excellent statistical analysis and high quality data evaluation.

Enzyme molecules cannot be loaded individually into the fL-sized reaction vessels. Therefore, separation depends on the random distribution of the enzyme molecules in the wells of the array. The Poisson distribution is a simple statistical method to determine the optimal enzyme concentration to maximize the number of wells occupied with a single enzyme molecule. In general, Poisson statistics describes the probability of a rare event in a large number of trials. When applied to the distribution of enzyme molecules in the wells of a fL-array, the probability *P*_μ_(*x*) that exactly *x* enzyme molecules are enclosed in a specific well can be calculated by Equation (8), where μ is the mean number of enzyme molecules per well:
(8)
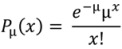


For example, at a ratio of one enzyme molecule per 20 reaction chambers, 95% of the reaction vessels are empty, 5% are occupied with a single enzyme molecule and only 0.1% of the wells contain more than a single molecule [11]. In order to further decrease the probability of multiple molecules per chamber, a stronger reduction of the enzyme concentration would be necessary resulting in an increasing number of empty wells. Consequently, when working with fL-arrays, a high degree of parallelization is mandatory. The large size of the array ensures that hundreds of enzyme molecules can be investigated in parallel even if only every twentieth reaction chamber is occupied.

Femtoliter chambers have an ideal size for single enzyme molecule investigation: They are small enough to isolate individual enzyme molecules and can accumulate a high number of product molecules in a short time. Additionally, they are big enough to hold a large excess of substrate molecules and ensure a constant substrate concentration over the whole course of the experiment. Only in this case the substrate turnover rate of individual enzyme molecules can be calculated accurately. A substrate concentration of 100 µM equals several millions of substrate molecules in a fL volume. β-Galactosidase catalyzes the hydrolysis of glycosidic bonds in β-galactopyranosides. The enzyme is both stable and shows a high turnover rate of 100 to 1000 substrate molecules per second, which leads to an average substrate depletion of less than 10% over the course of several minutes. For these reasons, it has repeatedly been used as a model enzyme for single molecule analysis [11,62,89].

## 4. Femtoliter Arrays in Optical-Fiber Bundles

**Figure 4:**
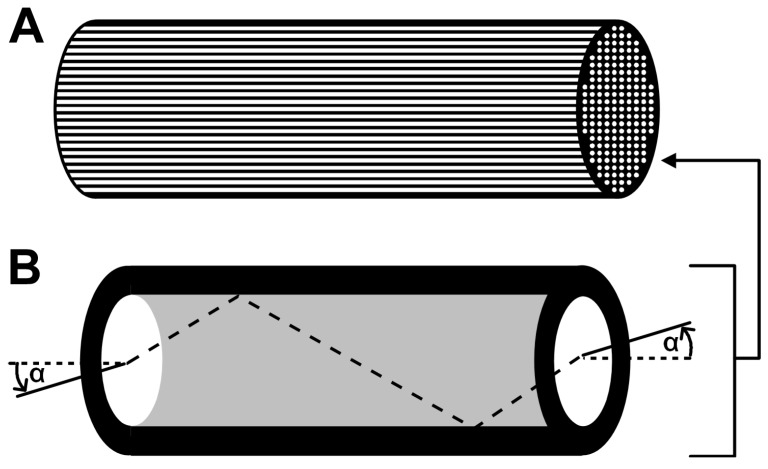
Femtoliter array on an optical-fiber bundle. (**A**) An optical-fiber bundle usually consists of several thousands of individually addressable fibers that are fused into a common cladding material; (**B**) Due to the different refractive indices of cladding material (black) and core, light propagates along the entire fiber length by total internal reflection. Schematic representation reprinted with permission from Wiley [94], © 2007.

Microwell arrays in optical-fiber bundles were first developed in the mid 1990s in the laboratory of David Walt [93]. Over the years the arrays were optimized and used for many bioanalytical applications. Optical-fiber bundles consist of hundreds to several thousands of individual glass fibers that are bundled, melted and fused into one unity [88]. Each fiber is composed of two different types of glass with different refractive indices. A core with diameters between 2 and 20 µm is surrounded by a common cladding material of lower refractive index than the core material (Figure 4). Light that enters the waveguide within a critical angle α is transmitted along the fiber by total internal reflection over long distances without severe attenuation [88]. The core material of the fibers can be etched selectively to form arrays of homogenous fL reaction chambers. The microwells are arranged in very high density arrays of about 25,000 mm^−2^. The fL reaction vessels on one end of the optical-fiber bundle are loaded with a fluorescent sample. The other end is connected to an epi-fluorescence microscope where the incoming light is filtered and detected by a sensitive CCD camera. Excitation light is launched into the entire array. The emission light is transmitted through individual cores at the bottom face of the microwells. Each fiber provides the signal from the reaction chamber to which it is linked. In this way, a multitude of individual reaction vessels can be investigated in parallel. [94] Typical optical-fiber bundles applied in single molecule enzymology contain about 50,000 fibers with an overall diameter of 1.5 mm [87].

Over the years optical-fiber bundle arrays were applied in many different single molecule enzymology studies. In a proof of principle experiment, individual molecules of β-galactosidase were enclosed in a homogeneous fiber bundle array together with an excess of resorufin-β-d-galactopyranoside (RDG) [11]. The microwells were sealed mechanically with a silicone gasket to ensure a constant reaction volume and to prevent evaporation or an exchange of solution between the wells during the measurement. β-Galactosidase hydrolyzes the fluorogenic substrate RDG to highly fluorescent resorufin, which can be monitored by wide-field fluorescence microscopy. Monitoring of a large population of β-galactosidase molecules revealed discrete and long-lived substrate turnover rates for individual β-galactosidase molecules. The broad activity distribution within an enzyme population (static heterogeneity) can be attributed to different conformational states and is consistent with previous single molecule studies [74,95,96].

**Figure 5:**
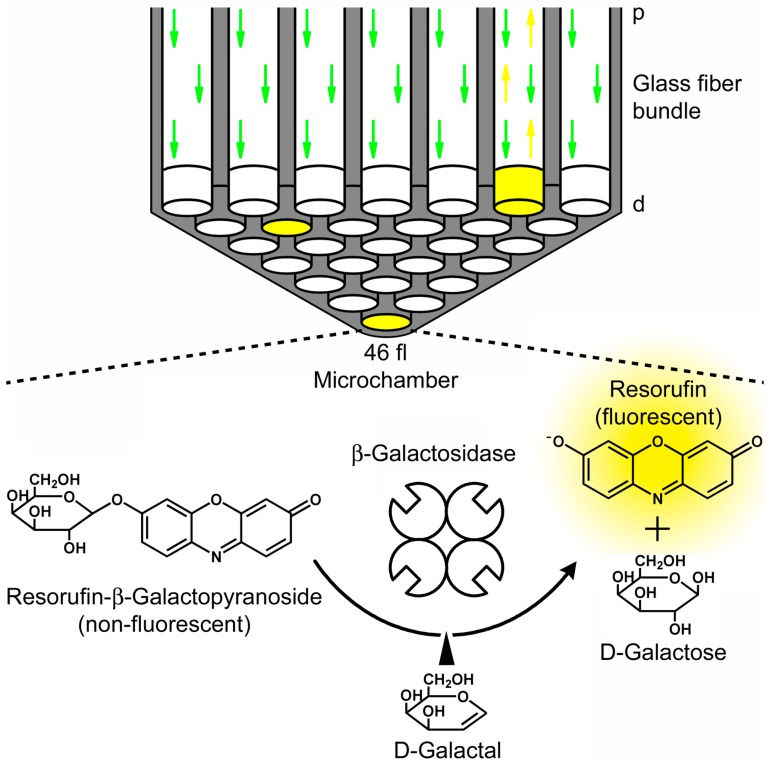
Investigation of competitive enzyme inhibition in optical-fiber bundle arrays. The fL-array consists of 50,000 wells defining a volume of 46 fL that are etched homogeneously into the distal end (**d**) of each fiber. Individual molecules of β-galactosidase together with the non-fluorescent substrate resorufin-β-d-galactopyranoside are enclosed in the containers. The substrate turnover into fluorescent resorufin is monitored through the proximal side (**p**) of the fiber bundle by fluorescence microscopy. The substrate turnover is inhibited when the slow-binding inhibitor d-galactal is binds to the enzyme. Schematic representation reprinted with permission from the National Academy of Sciences, USA [26], © 2007.

β-Galactosidase was further used for the first single-molecule investigation of competitive enzyme inhibition in optical-fiber bundle arrays (Figure 5) [26]. Binding and release rates of the slow-binding inhibitor d-galactal from single β-galactosidase molecules were observable as stochastic events. These measurements revealed a one-step transition from no enzyme activity to the highest state of activity. The lack of intermediate activity states is indicative of a cooperative inhibitor release from all four monomers. The rate constants determined from stochastic inhibitor binding and release experiments were consistent with bulk enzyme kinetics. This work was further expanded [27] by comparing the activity of β-galactosidase in the presence of d-galactal and *N*-*p*-bromobenzylaminohydroxy methylcyclopentanetriol (NpBHC). The inhibitor-release kinetics of these inhibitors was fundamentally different: while d-galactal release from individual molecule of β-galactosidase was cooperative [26], NpBHC was released sequentially from the four enzyme subunits. It was shown that d-galactal, the less potent inhibitor, has a stronger effect on the enzyme conformation than NpBHC. An autocorrelation analysis of the substrate turnover rates revealed that the inhibitor exchange rates of d-galactal and NpBHC are inversely correlated to the respective substrate turnover rates. A multiple substrate experiment additionally showed that the active site can make a selection for either the substrate or the inhibitor, while in substrate-substrate competition the active site is not selective for either substrate.

Recently, the Walt group investigated heating effects on the activity of β-galactosidase [97]. Upon heating the individual enzyme molecules switched between different activity-states resulting from conformational changes. The activity changes were random and did not correlate with the enzyme’s original activity. Consequently, the static heterogeneity within an enzyme population is related to the presence of different stable conformations and individual β-galactosidase molecules possess numerous stable activity states that can be interconverted upon exposure to thermal energy [97].

Single enzyme molecule experiments of horseradish peroxidase (HRP) enclosed in optical-fiber bundle arrays revealed a ten times lower substrate turnover rate for HRP at the singe-molecule level compared to the bulk experiment [98]. This phenomenon was explained by the complex redox mechanism of HRP catalysis that involves two separate steps of product formation and the generation of radical intermediates. The high surface to volume ratio for experiments performed in fL-arrays increases the probability of potential side reactions of the highly active radical intermediates. The two-step reaction mechanism not only affects single molecule studies on HRP but also bulk experiments at low substrate turnover rates [98].

Evaporation of water from microwells is a significant problem when working with arrays of ultra-small reaction vessels [89,90,99]. Instead of sealing the fiber bundle arrays mechanically by a silicone gasket, oil-sealing of the fL-sized reaction chambers was explored to ensure a tight enclosure and avoid evaporation of the aqueous solution [100]. The fiber bundle surface was rendered hydrophilic by acid etching. Contact printing was applied subsequently to selectively modify the surface of the cladding material between the microwells with a hydrophobic silane, while the inner surface of the wells was kept hydrophilic. The wells of the fiber bundle array were then filled with individual enzyme molecules and the corresponding fluorogenic substrate by immersing the modified fiber in the aqueous sample solution for a few minutes. After removing excess solution, the end of the fiber was covered with a drop of fluorinated oil to seal the reaction chambers and prevent evaporation.

In addition to basic research, the enzymatic turnover of single enzyme molecules in optical-fiber bundle arrays has been employed for implementing a single molecule ELISA [101,102]. In this case, the enzyme is used as a reporter for the detection of other analyte molecules. The enzymatic signal amplification enables the detection of analytes with high sensitivity. In traditional microtiterplate immunoassays, the reaction volume of about 100 µL is far too large for the detection of individual analyte molecules. Essentially millions of fluorophor molecules are required to obtain a signal that can be detected against the comparatively high background scattering of solvent molecules. In the wells of a fL-array, however, the detection volume is decreased by a factor of 10^10^, which enables the design of so-called single-molecule immunoassays. In a proof of principle experiment, single molecules of streptavidin-labeled β-galactosidase were bound to a biotin-derivatized reaction vessel surface [103]. After sealing, the single enzyme molecules were detected by monitoring the accumulation of fluorescent product. This technique has been further developed to enable the detection of varying biological analytes such as proteins [103] or DNA [104]. In single-molecule immunoassays, the analyte concentration can be determined by counting the chambers that light up if a single reporter enzyme molecule turns over the fluorogenic substrate. In contrast to traditional analogue immunoassays, the signal generated by a single enzyme molecule in a femtoliter well will never fall below the detection limit. The progress of protein detection in microwell arrays was recently highlighted [93].

**Figure 6:**
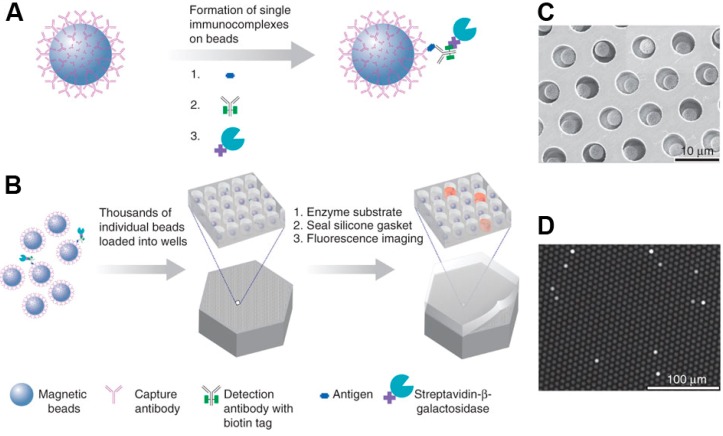
Digital immunoassay in optical-fiber bundle arrays. (**A**) Microbeads covered with capture antibodies are incubated with the sample containing the target protein of interest. A biotinylated detection antibody and a streptavidin-labeled β-galactosidase are used to label the captured protein. An excess of beads compared to the number of target protein molecules is added such that most beads contain zero molecules while some beads contain one bound protein according to Poisson statistics. Only beads containing a target protein molecule carry a β-galactosidase label; (**B**) The beads are isolated in wells of the fiber bundle array where the substrate turnover is detected by fluorescence microscopy; (**C**) Scanning electron image of a small section of the fiber bundle array after bead loading; (**D**) The fluorescence image of the fL-array demonstrates the generation of fluorescent product in some of the wells that contain a bead with protein-immuno-complex. The protein concentration is correlated to the number of “active” chambers. Schematic representation reprinted with permission from Macmillan Publishers [105], © 2010.

A single-molecule ELISA has been commercialized by Quanterix Corporation (Lexington, MA, USA) for detecting proteins in blood at femtomolar or even subfemtomolar concentrations (Figure 6) [105,106,107]. For this purpose, the proteins of interest were pre-concentrated on microscopic beads that carried specific antibodies.

These immuno-complexes were labeled with a streptavidin-β-galactosidase conjugate responsible for the generation of a fluorescent product. For samples containing extremely low protein concentrations, the ratio of protein molecules to beads was small and the percentage of beads that contained a labeled immuno-complex followed a Poisson distribution. The beads were loaded into the 50-fL reaction chambers of a fiber bundle array and detected by fluorescence microscopy. The digital ELISA enabled the identification of immune-complexes on beads at concentration of 10^−19^ M. Clinically relevant proteins such as prostate specific antigen (PSA) and tumor necrosis factor-α (TNF-α) could be detected routinely in serum at subfemtomolar concentrations, which is far below the detection limit of conventional immunoassays. The bead array can also be used for detecting single molecules (digital readout) and ensembles of molecules (analog readout) simultaneously [108]. At low protein concentrations (enzyme-label to bead ratio <1.2), the beads carry either zero or a low number of enzymes and the protein concentration can be determined by counting the number of fluorescent beads (digital readout). At higher analyte concentrations, a high number of enzyme labels are attached to each bead and the average number of enzyme labels per bead can be quantified from the relative intensity of the fluorescence signal (analog readout). By combining digital and analog protein detection, an immunoassay for PSA was developed with a linear response over approximately four orders of magnitude [108].

The fiber bundle microarrays have recently been replaced by fL-arrays integrated in an enclosed microfluidic device fabricated in a thermoplastic polymer [109]. The thermoplastic device was generated by an injection molding technique. It consists of well arrays and fluidic channels that were fabricated in separate planar cyclic olefin polymer (COP) polymeric parts. Both parts were joined and bonded to form an integrated assembly. The polymer arrays were oil-sealed in an integrated microfluidic device that facilitated the isolation of single beads in the fL-wells and provided further advantages such as low-cost manufacturing, or the possibility to establish fully automated single-molecule array systems.

A multiplexed single molecule immunoassay was developed by using fluorescently encoded beads. [110]. Each type of antibody was bound to a subset of beads that could be identified by a distinct fluorescent signature. After incubation with an analyte sample and an antibody-enzyme reporter, each type of protein was immobilized on a distinct subset of beads. The bead-protein complexes were loaded into the wells of a thermoplastic fL-array and a single antibody-enzyme conjugate generated a signal that was recorded by fluorescence microscopy. Four different proteins (TNF-α, IL-6, IL-1α and IL-1β) were detected simultaneously in human plasma at subfemtomolar concentrations [110]. Additionally, the concentration of each protein could be determined from the average number of enzyme molecules per bead given by the signal intensity of the fluorescence images.

In addition to single enzyme molecule analysis, the Walt group has also employed optical-fiber bundles for establishing DNA arrays [111,112,113]. For example, optically encoded microspheres confined in fiber-optic arrays were applied for the simultaneous detection of six biological warfare agents [111]. The array enabled the correct identification of bacterial target DNA with a detection limit of 10 fM. In a similar way, chromosomal DNA from *Salmonella spp.* and ribosomal RNA from several harmful algal bloom species could be detected [112,113]. Optical-fiber bundle arrays were further applied to isolate and investigate individual cells. Yeast [114], bacteria [115,116,117] and mammalian cells [118,119] have been enclosed separately in the wells of an optical-fiber bundle array together with a solution of the required nutrients. The single-cell studies provided new details regarding cellular processes and enabled the functional screening of biochemically active reagents. Recently, Vajrala *et al.* [120] investigated individual mitochondria in the wells of an optical-fiber bundle array. Utilizing the fluorescence of NADH, the metabolic status of individual mitochondria at varying respiratory states was monitored by fluorescence microscopy.

## 5. Femtoliter Arrays Molded in PDMS

Soft lithography is another established method for the fabrication of arrays of fL-sized reaction containers. Soft lithography is a low-cost and effective method based on replica molding for the generation of microstructures [121,122]. A master mold is applied as a template to cast complementary structures in elastomers such as polyurethanes, polyimides and most commonly poly(dimethylsiloxane) (PDMS). PDMS is well-suited for the fabrication of bioanalytical assay systems as it is nontoxic and oxygen-permeable. Furthermore, it is transparent and can be easily patterned into minute structures. For example, Rettig *et al*. [123] and Sasuga *et al*. [124] isolated individual cancer cells in thousands of PDMS microwells defining a picoliter (pL) volume. They used fluorescence imaging to test the cells for vitality [123] or to determine intracellular protein concentrations and enzymatic activities [124].

**Figure 7:**
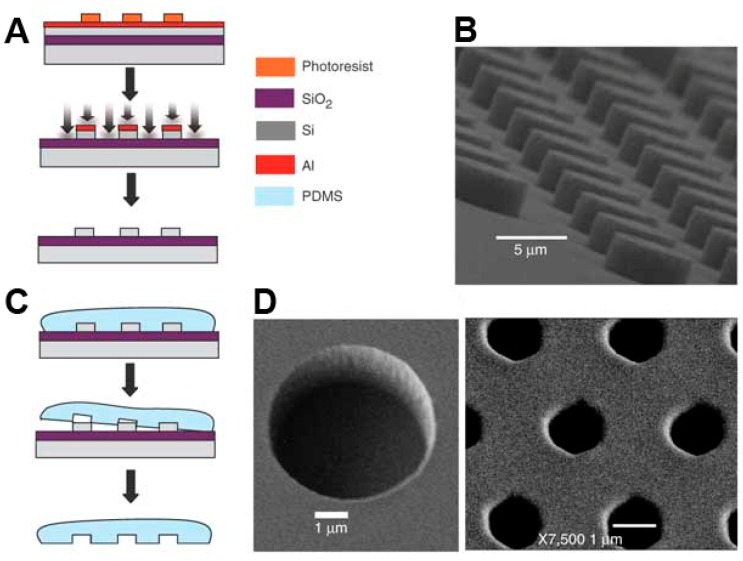
Femtoliter array molded in PDMS. (**A**) Fabrication of the template: a silicon wafer is covered with an aluminum mask and patterned by photolithography; (**B**) Scanning electron microscopy (SEM) image of the silicon template demonstrating the regular array of homogeneous cylindrical shapes; (**C**) Liquid PDMS was poured on the teflon-coated mold, polymerized at high temperature and finally peeled off; (**D**) SEM images of the PDMS fL-array. Schematic representation reprinted with permission from Macmillan Publishers [89], © 2005.

Single enzyme molecules were first analyzed in fL arrays of PDMS by Rondelez and colleagues [89]. A silicon master stamp patterned by photolithography was used to mold series of identical PDMS sheets with integrated arrays of 30 fL reaction containers (Figure 7). The containers were sealed via PDMS adhesion to a glass coverslip under mechanical pressure. In a proof of principle experiment, the hydrolysis of fluorescein-di-β-d-galactopyranoside (FDG) by single molecules of β-galactosidase was monitored using wide-field fluorescence microscopy. The PDMS array was further applied in several studies to investigate the biomechanical processes involved in ATP synthesis and hydrolysis, catalyzed by the enzymes F_1_-ATPase and F_0_F_1_-ATP synthase [125,126,127,128]. The rotation of the motor enzyme F_1_-ATPase is mechanochemically coupled to ATP generation and hydrolysis. F_1_-ATPase was labeled with a magnetic bead and attached to the surface of a glass slide. The glass was covered with a fL array molded in PDMS such that single F_1_-ATPase molecules were isolated in the wells of the array. The magnetic bead was rotated clockwise with magnetic tweezers to induce ATP synthesis. After the magnetic field was turned off, the molecular motor rotated in an anticlockwise direction at a speed proportional to the amount of synthesized ATP [125]. The assay setup was employed repeatedly to clarify mechanistic details of the F_1_-ATPase and F_0_F_1_-ATP synthase mediated ATP synthesis and hydrolysis [126,127,128].

Arata *et al*. [129] developed a PDMS fL-array combined with an integrated on-chip microreactor and microheater that enabled the measurement of enzymatic activity at high temperatures. The microheater together with a thermosensor consisted of nickel-structures integrated in a glass plate via photolithographic Ni-etching. The microheater chip was attached to a patterned PDMS-array sheet via adhesion forces. The on-chip microheater PDMS array system was applied to investigate the temperature dependency of the β-galactosidase activity. β-Galactosidase survived short-time exposure to high temperatures. Additionally, the enzyme activity was found to be about four times higher at 60 °C than at room temperature. Temperature effects on the activity of single β-galactosidase molecules were further explored in optical-fiber bundle arrays as described in the previous chapter [97].

Single α-chymotrypsin molecules were isolated and investigated in arrays of 4.2-fL reaction chambers molded in PDMS [130]. The protease activity of individual α-chymotrypsin molecules was monitored using a protein-dye conjugate consisting of casein labeled with a large number of self-quenched fluorophores. After proteolysis by chymotrypsin, the fuorophores were spatially separated, which led to a 50- to 100-fold increase of the fluorescence signal. Consistent with other studies on single enzyme molecules, individual α-chymotrypsin molecules exposed a heterogeneous activity within the enzyme population.

Microfluidic methods play an important role in delivering fluids to fL wells. Jung *et al*. [131] combined an array of homogeneous PDMS microwells (4.4 µm diameter and 6.5 µm height, defining a volume of 100 fL) with a microfluidic device to control the initiation of the enzymatic reaction. [131] In a proof of principle experiment, the reactants β-galactosidase and resorufin-β-d-galactopyranoside were introduced from separate inlets and combined in less than 100 ms in a mixing channel. The homogeneous enzyme-substrate mixture was then enclosed in the wells of the PDMS fL array by using a glass coverslip under hydraulic pressure. In this microfluidic system, single enzyme molecule kinetics could be monitored within milliseconds after mixing enzyme and substrate.

Another method to generate high-density fL arrays within microfluidic channels in PDMS was presented by Ota *et al*. [132]. Many small reaction chambers (5 µm in diameter and 6 µm in depth) were formed in the walls of a main channel by PDMS molding. When aqueous solutions and organic solvents were subsequently infused into the channel, aqueous droplets were confined in the chambers by the organic solvent. β-Galactosidase in the droplets catalyzed the hydrolysis of fluorescein-di-β-d-galactopyranoside (FDG) to fluorescent fluorescein, which was monitored in parallel in about 300 reaction chambers by fluorescence microscopy. The differences in the fluorescence signal were proportional to the enzymatic activities in each reactor and showed a clear quantization indicating the presence of zero, one, two or three enzymes in the respective microchambers according to Poisson statistics.

**Figure 8:**
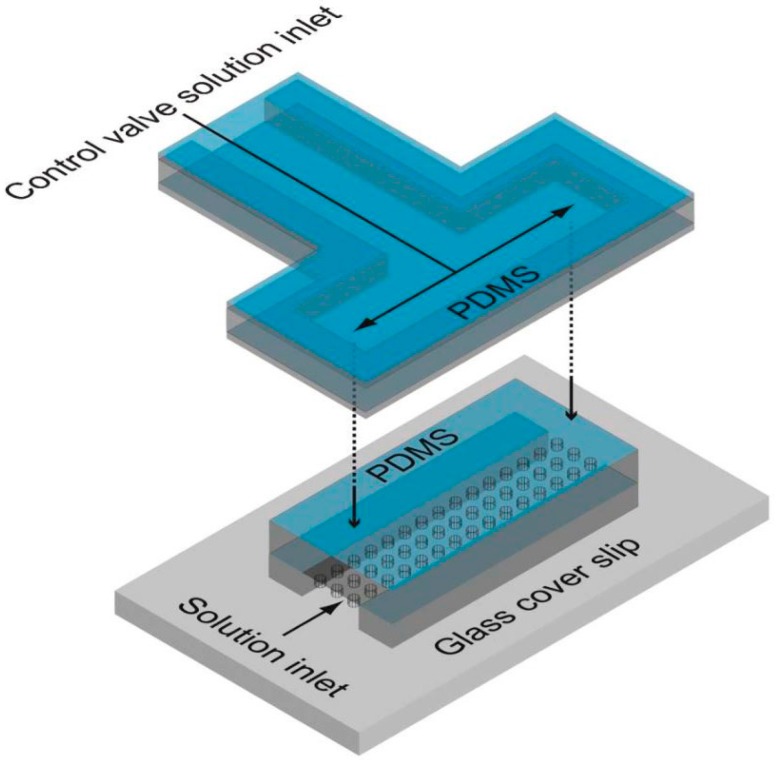
Microfluidic device with integrated fL-volume chambers molded in PDMS. The microwell array is hydraulically actuated with a control valve in a second PDMS layer. The mobility of individual fluorescent molecules can be investigated in the microfluidic PDMS array. Modified schematic representation reprinted with permission from the Royal Society of Chemistry [133], © 2013.

Microfluidic PDMS systems have not only been applied to investigate single enzyme molecules. Recently, Fowlkes and colleagues [133] studied the mobility of individual fluorescent molecules in a microfluidic device with sealable fL-volume reaction chambers (Figure 8) by fluorescence correlation spectroscopy. The microfluidic system was formed in PDMS and fabricated by multilayer soft-lithographic techniques. Arrays of homogeneous reaction chambers in 160 µm wide microchannels were replicated from master templates. Control valves were integrated into a second PDMS layer by micromolding. Later the valve system was aligned and bonded to the chamber/channel system by additional curing. Finally, holes were punched through the valve system for inlets and outlets and the combined replica was bonded to a PDMS-coated glass coverslip by plasma treatment.

## 6. Femtoliter Arrays Fabricated in Glass Slides by Photolithography

Topographically patterned surfaces in hard materials such as glass coverslips are usually fabricated by photolithography or particle beam lithography [134]. Large arrays of homogeneous fL-wells were microfabricated by photolithography into the surface of fused silica slides [95]. Standard etching techniques were applied to generate cylindrical wells, 8 µm in diameter and 4 µm deep, defining a volume of 135 fL. The wells were loaded with individual enzyme molecules and an excess of substrate by ultrasonication in combination with vacuum degassing. A very thin fused silica coverslip was used to seal the liquid-filled reaction chambers. In this array format the enzymatic activity of single molecules of lactate dehydrogenase (LDH-1) was monitored over time [95]. LDH-1 catalyzes the redox reaction of lactate and nicotinamide adenine dinucleotide (NAD^+^) resulting in pyruvate and fluorescent NADH. The enzyme-catalyzed redox-process was compared to the Os^III^-catalyzed redox reaction of Ce^IV^ and As^III^ to fluorescent Ce^III^ and As^V^. The formation of the fluorescent product was monitored by wide-field fluorescence microscopy and a CCD camera. The activity distribution observed for single metal ions was considerably narrower than the broad distribution obtained for individual enzyme molecules. The broad activity distribution within the LDH-1 population was attributed to varying protein conformations which distinguishes enzymatic catalysis from metal ion catalysis [95].

Recently, arrays of fL droplets were generated on a hydrophilic-in-hydrophobic micropatterned surface [69,135,136]. A hydrophobic carbon-fluorine polymer was spin-coated on a clean coverglass. Photolithography and reactive ion etching was conducted subsequently to expose the hydrophilic SiO_2_ surface. The hydrophilic-in-hydrophobic micro-patterned coverglass was covered with an aqueous solution. Fluorinated oil, which has a higher density than water, was then flowed into the aqueous solution near the micro-patterned surface. The hydrophobic surface was covered with oil, while the hydrophilic glass surface retained the aqueous solution in the form of many homogeneous droplets. In this way, more than 10^6^ dome-shaped droplets were prepared simultaneously. Individual β-galactosidase molecules were enclosed in the droplets together with the fluorogenic substrate fluorescein-di-β-d-galactopyranoside to measure the activity of single enzyme molecules. The fluorescence images of the droplets were recorded on a confocal microscope.

In connection with previous experiments conducted in PDMS arrays [125,126,127], Noji and colleagues [69] investigated the kinetic parameters of the rotary motor protein F_1_-ATPase in the hydrophilic-in-hydrophobic array system. Streptavidin-coated polystyrene beads were attached to the rotor γ subunit of F_1_ to visualize the enzyme’s rotation. The stator α_3_β_3_ ring of F_1_ was immobilized on the glass and the surface was passivated, preventing non-specific binding of the probe-beads. Subsequently, the bead-enzyme suspension containing ATP was injected. Phase-contrast images of the rotating beads were recorded and the rotation rate depending on the ATP-concentration was calculated.

**Figure 9:**
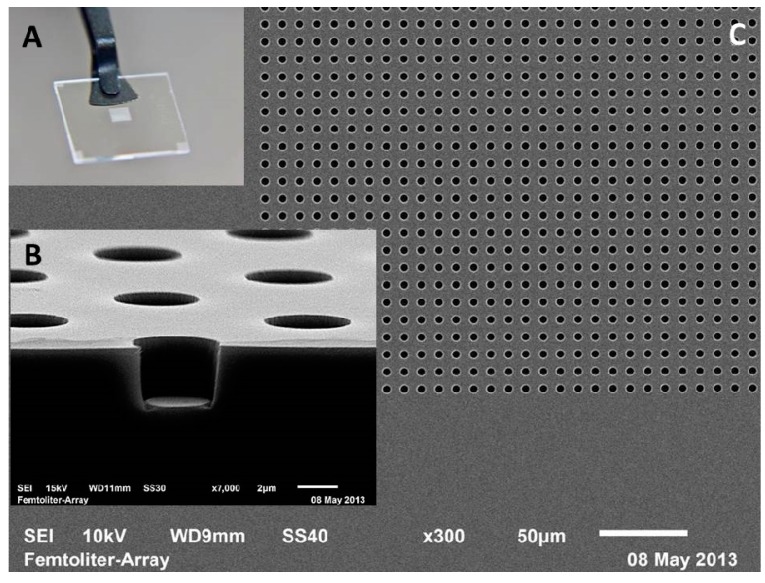
Femtoliter array etched into the surface of a fused silica slide by photolithography. (**A**) Photograph of the fL-array located in the center of a fused silica slide; (**B**) Side view of the array highlighting one cylindrical reaction chamber with a diameter of 4 µm and a depth of 3.5 µm, which defines a volume of 40 fL; (**C**) SEM image showing a lattice of regularly arranged and homogeneous fL-wells.

In our group, we have prepared homogeneous arrays of 62,500 fL-sized reaction vessels etched into the surface of fused silica slides to enclose single enzymes molecules. For the fabrication of the glass arrays, a positive photoresist was spin-coated on a fused silica wafer. A chrome mask was vacuum-contacted to the wafer and exposed to UV light. The wafer with the patterned photoresist was subjected to reactive ion etching to structure the fused silica surface. On a four-inch wafer, 21 homogeneous arrays with an edge length of 2.5 × 2.5 mm positioned in the center of 15 × 15 mm glass slides were generated in parallel. The arrays consisted of 250 × 250 = 62,500 cylindrical wells with a diameter of 4 µm and a depth of 3.5 µm defining a volume of approximately 40 fL. The dimensions of the fL wells and the reproducibility of fabrication were confirmed by scanning electron microscopy (SEM) (Figure 9). For single-molecule enzymology, a dilute enzyme solution and an excess of a fluorogenic substrate was pipetted onto the array and sealed with a PDMS gasket. The fluorescent product of the enzyme reaction was then monitored over time by wide-field fluorescence microscopy. In the fused silica array system, we investigated the oxidation of the fluorogenic substrate Amplex Red to fluorescent resorufin by hundreds of individual horseradish peroxidase (HRP) molecules [92]. The fluorescence excitation and the detection scheme were optimized to minimize the photooxidation of Amplex Red and photobleaching of resorufin.

We demonstrated the presence of long-lived kinetic states of individual HRP molecules that are different for each HRP molecule in the enzyme population. Additionally, we confirmed previous results in optical-fiber bundle arrays [98] which showed a 10-fold lower product formation in fL-sized chambers compared to a bulk solution because of a particular two-step redox mechanism of HRP.

**Figure 10:**
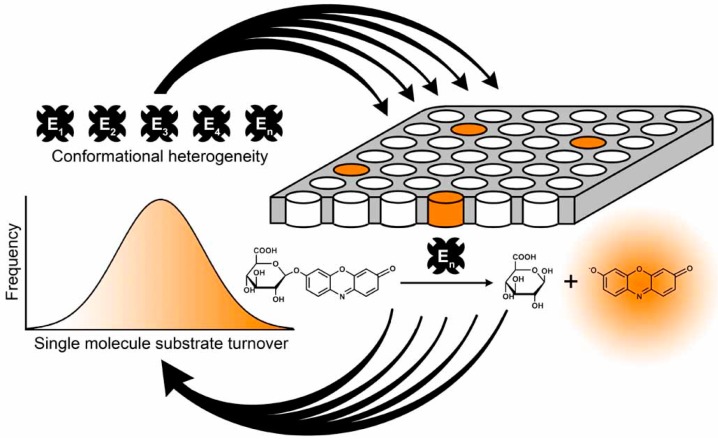
Single molecule analysis of β-glucuronidase (GUS) in the wells of a fl-array. Individual molecules of a conformationally heterogeneous enzyme population (E_1_–E_n_) are isolated in a fused silica array consisting of 62,500 homogeneous fl wells on the surface of a fused silica slide. Individual GUS molecules hydrolyze the non-fluorescent substrate resorufin-β-d-glucuronide to fluorescent resorufin which is recorded by fluorescence microscopy. The substrate turnover of hundreds of individual GUS molecules is recorded in parallel in separate fl chambers and assembled as histograms to demonstrate the activity distribution within the enzyme population. Schematic representation reprinted with permission from the American Chemical Society [137], © 2014.

We further applied the fused silica fL-array to investigate enzyme evolution of β-glucuronidase (GUS) at the single molecule level [137]. Like β-galactosidase, GUS catalyzes a simple hydrolytic reaction. We separated several hundred single GUS molecules in the wells of fused silica fL-arrays and observed their individual substrate turnover rates in parallel by fluorescence microscopy (Figure 10). Individual GUS molecules display long-lived and individually different activity states, while the mean activity is consistent with Michaelis-Menten kinetics. Compared to wild-type GUS, *in vitro* evolved GUS displays a much broader activity distribution among individual enzyme molecules, which can be attributed to a broad conformational heterogeneity within the population of evolved enzymes. A broad distribution of conformations enables the turnover of different substrates, which broadens the substrate specificity of an enzyme population [138,139,140,141].

Femtoliter arrays in glass slides have not only been used for single molecule enzymology. Iino *et al*. [142,143] applied the design of the fl-droplet array, described above [69], for the development of a single-cell drug efflux assay in individual cells of *Escherichia coli*. One wild-type and two different mutant strains of *E. coli* cells with an efflux-pump-gene deletion were mixed with fluorogenic fluorescein-di-β-d-galactopyranoside (FDG) and isolated in a microdroplet array. Upon entering the *E. coli* cell, FDG is hydrolyzed to fluorescent fluorescein by β-galactosidase. In wild-type cells, FDG was successfully pumped out of the cells by the intact multicomponent pump complex and no fluorescence was detected. In contrast, the pump-complex was not expressed correctly in the mutant cells.

Therefore, the imported FDG was transformed to fluorescein which could be detected by fluorescence microscopy. Iino *et al*. also investigated the properties of an efflux pump inhibitor on the drug efflux activity of the *E. coli* cells, which showed a concentration-dependent inhibitory effect [142].

Single-molecule investigations on DNA-hybridization have been conducted in fl-arrays fabricated in fused silica coverslips [90]. The so-called “Dimple Machine” consists of a fused silica coverslip containing multiple arrays of nanofabricated circular drops or dimples fabricated by electron-beam lithography. Typically, four dimple arrays were written on one coverslip and each array consisted of 900 circular dimples with diameters between 70 nm and 1.3 µm and a depth of 200 nm arranged in a square lattice with 4 µm spacing. The dimples were loaded with a solution containing two strands of fluorescently labeled single-stranded DNA molecules and reversibly sealed with a pneumatically actuated, structured PDMS lid. DNA hybridization was monitored by co-localization and fluorescence resonance energy transfer (FRET) [90]. The operation of array sealing and opening was fully automated and allowed for the frequent repetition of the “trap-measure-refresh” cycle.

**Figure 11:**
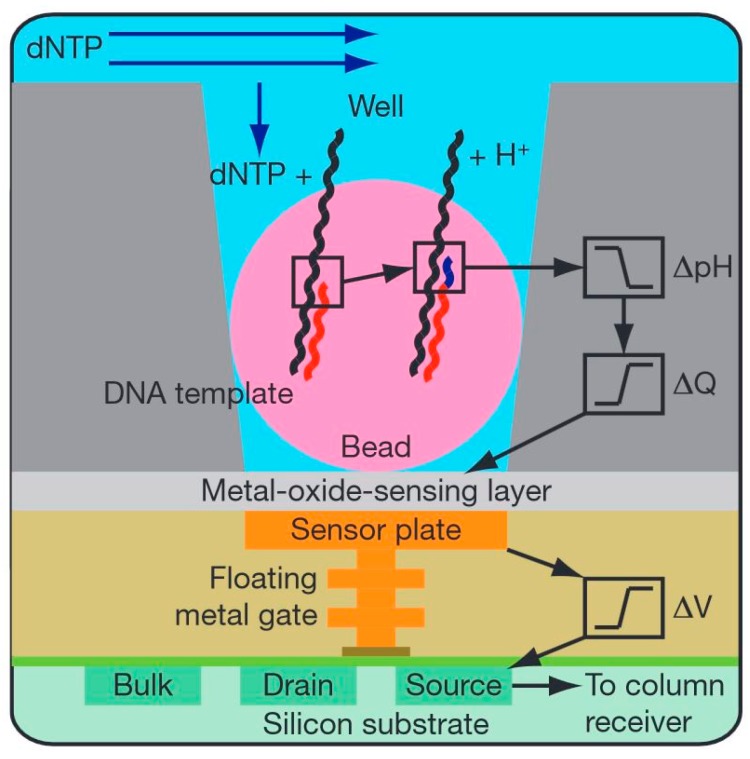
Schematic representation of the architecture of a fL chamber for non-optical signal readout. Simplified drawing of a microwell containing a bead with DNA-template and the underlying sensor and electronics: Protons released upon nucleotide (dNTP) incorporation in DNA strands change the pH (ΔpH) in the well. The pH-shift induces a change in surface potential of the metal-oxide-sensing layer, and a change in potential (ΔV) of the source terminal of the underlying field-effect transistor. Schematic representation reprinted with permission from Macmillan Publishers [144], © 2011.

Recently, Rothberg *et al*. [144] presented an array system of homogeneous fL-sized reaction containers that allowed for a non-optical signal readout. The ion-sensitive-field-effect-transistor (ISFET) sensor enabled direct DNA-sequencing without any optical components. The circuit consists of a large array of approximately 200 individual sensor elements, each with a single floating gate connected to the underlying ISFET (Figure 11). Each sensor element independently recorded the protons released upon nucleotide incorporation during DNA synthesis.

The natural nucleotides were confined in wells of 3.5 µm diameter, fabricated in a 3 µm-thick dielectric layer on top of the ISFET by a chemical etching process. The all-electronic detection system simplifies signal recording, reduces the cost of the sequencing instrument and combines high-speed addressing and readout.

## 7. Challenges and Opportunities for Analyzing Single Enzyme Molecules in Femtoliter Volumes

The analysis of individual enzyme molecules in femtoliter volumes is still limited compared to the wealth of enzymatic studies performed in bulk solution. One reason for this is the limited availability of fluorogenic substrates that are stable and completely non-fluorescent and become highly fluorescent only after enzymatic catalysis. The generation of new, stable fluorogenic substrates or the development of alternative readout schemes is therefore essential. Another problem of fluorogenic substrates relates to their mode of conversion. Many fluorogenic substrates contain two enzymatically cleavable bonds conjugated to one fluorophore. Consequently, the enzymatic catalysis proceeds in two steps. For example, fluorescein-di-β-d-galactopyranoside (FDG) is frequently used as a model substrate for monitoring the activity of single β-galactosidase molecules in femtoliter volumes. The quantum yield of the mono-substituted fluorescein intermediate, however is very low and strongly depends on the substituent. [145,146,147,148] The two-step reaction results in sigmoidal kinetic curves that are very difficult to analyze. Consequently, resorufin-β-d-galactopyranoside that contains only a single cleavage site for β-galactosidase greatly simplifies the kinetic analysis. Similarly, there are two cleavage sites in most fluorogenic substrates for proteases that catalyze the hydrolysis of peptide bonds [149]. To circumvent this limitation, Terentyeva *et al*. [150] developed fluorogenic substrates for α-chymotrypsin containing only one cleavable peptide bond and compared their hydrolysis with conventional substrates containing two cleavage sites. The study revealed that the double-substituted substrate analogues yield kinetic parameters that are significantly different from those obtained from mono-substituted substrate analogues. Progress in single molecule analysis thus also relies on the systematic development of new fluorogenic substrates. On the side of the enzyme, it must be taken into account that most enzymes are multimers. Consequently, the substrate turnover results from the activity of several catalytic sites, which complicates the kinetic analysis.

Finally, it is essential to address possible surface reactions that are a consequence of the large surface to volume ratio in femtoliter wells. For example, non-specific binding of the enzyme can be avoided by adding an excess of blocking reagents that are not involved in the reaction [11]. Furthermore, we have found that the signal generation of horseradish peroxidase enclosed in a femtoliter well was ten times lower than in bulk reaction [92,98]. This can be explained by a two-step reaction mechanism which leads to the formation of a radical intermediate that can also react with the well surface instead of forming the fluorescent product. New solution additives and surface chemistries for surface passivation are needed to reduce non-specific protein binding.

In analytical applications such as single molecule ELISAs, one has to consider that even a large array can hold only a limited amount of probe volume. For example, a femtoliter array of 100,000 wells each defining a volume of 50 fL can only hold a probe volume of 5 nL. Consequently, a pre-concentration step is essential to probe a larger volume. This can typically be achieved by implementing bead-based microwell arrays [56,88,93]. Additionally, microfluidic devices can be used for analyte sampling and delivering fluids to the femtoliter arrays. Recently, several microfluidic systems for single molecule applications have been reported. [131,132,133] Furthermore, to make single enzyme analysis applicable in clinical studies, multiple targets need to be measured in parallel in one sample. Rissin *et al*. [110] recently reported the successful operation of multiplexing in bead microwell arrays. Introduction of multiplexing is an important step towards the implementation of single molecule analysis as a standard method in biomedical applications.

## 8. Conclusions

The confinement of bioanalytical reactions in arrays of uniform, fl-sized reaction chambers has disclosed new mechanistical aspects of biochemical processes [151]. Many established single-molecule experiments are limited by the low number of enzyme molecules that can be monitored in parallel in each experiment. By separating individual biomolecules in large arrays of homogeneous microcompartments, one can perform thousands of analytical measurements in parallel. In this review, we have introduced various methods for the generation and application of large arrays of uniform reaction chambers in single-molecule enzymology. Different etching techniques can be applied to fabricate fl wells on optical-fiber bundles or in the surface of fused silica coverslips. Alternatively, fL wells in PDMS or cyclic olefin polymer (COP) can be molded on structured silicon templates.

The analysis of enzymes at the single-molecule level has provided us with new information about enzyme kinetics and conformational characteristics that cannot be obtained from bulk experiments. Due to the highly parallel array schemes presented in this review, modern single-molecule experiments provide excellent statistics on the activity distribution in an enzyme population. The possibility to analyze many enzymes simultaneously also simplifies the investigation of enzyme reactions under different reaction conditions. Femtoliter arrays can be employed for fundamental research on single molecule kinetics as well as for analytical measurements. For example, we presented single molecule sensing applications that enable the digital readout of very low analyte concentrations in complex matrices. Altogether, the continuing development of novel methods for generating large arrays of homogeneous microcompartments and the constant quest for improvement guarantee fast progress in the field of single-molecule bioanalysis in fL volumes in the near future.
